# Detection of Influenza D Antibodies in Dogs, Apulia Region, Italy, 2016 and 2023

**DOI:** 10.3201/eid3005.231401

**Published:** 2024-05

**Authors:** Claudia Maria Trombetta, Serena Marchi, Maria Giovanna Marotta, Ana Moreno, Chiara Chiapponi, Emanuele Montomoli, Gianvito Lanave, Michele Camero, Vito Martella

**Affiliations:** University of Siena, Siena, Italy (C.M. Trombetta, S. Marchi, M.G. Marotta, E. Montomoli);; VisMederi Research srl, Siena (C.M. Trombetta, E. Montomoli);; Istituto Zooprofilattico Sperimentale della Lombardia e dell’Emilia-Romagna, Brescia, Italy (A. Moreno, C. Chiapponi);; VisMederi srl, Siena (E. Montomoli); University of Bari, Valenzano, Italy (G. Lanave, M. Camero, V. Martella)

**Keywords:** Influenza, influenza D, influenza virus, viruses, dogs, seroprevalence, zoonoses, Italy

## Abstract

Dogs are known to be susceptible to influenza A viruses, although information on influenza D virus (IDV) is limited. We investigated the seroprevalence of IDV in 426 dogs in the Apulia region of Italy during 2016 and 2023. A total of 14 samples were positive for IDV antibodies, suggesting exposure to IDV in dogs.

Influenza D virus (IDV) was first identified from swine exhibiting influenza-like symptoms in 2011 in the United States (*1*). IDV RNA or antibodies have been detected worldwide in several animal hosts, including cattle, swine, small ruminants, camelids, and wild ungulates, although cattle are the main reservoir (*2*). 

Because of social interactions, dogs are known to occasionally transmit pathogens to humans. In addition to being susceptible to canine influenza A viruses, dogs seem susceptible to common human influenza viruses and to avian viruses. Antibodies to influenza C virus (ICV) have been reported, suggesting that dogs may be naturally infected by ICV (*3*). However, information on the susceptibility of dogs to IDV is lacking.

We estimated the seroprevalence of IDV in household adult dogs on a total of 426 serum samples collected in 2016 (n = 169) and in 2023 (n = 257) in the Apulia region, Italy. The samples were collected by veterinary offices either for presurgical evaluation (n = 361) or for routine analysis (n = 65). We tested the samples in duplicate by using a hemagglutination inhibition (HI) assay against 2 IDV strains, D/bovine/Oklahoma/660/2013 (D/660 lineage; (provided by Prof. Feng Li, University of Kentucky) and D/swine/Italy/199724–3/2015 (D/OK lineage; obtained from the European Virus Archive). We tested the HI-positive samples and a subset of negative samples by virus neutralization (VN) assay. We analyzed the IDV-positive samples collected in 2023 for the presence of ICV antibodies (human influenza C/human/Victoria/2/2012 virus; provided by Victorian Infectious Diseases Reference Laboratory, Melbourne, Victoria, Australia), although the 2016 samples could not be screened because of the limited residual volume (Appendix Figure 1). We considered an IDV antibody titer >10 a positivity cutoff for the HI and VN assays (*4*).

Our screening of the 2016 samples for HI (Table 1) resulted in 2 samples (2/169; 1.2% [95% CI 0.05%–4.5%]) testing positive for the D/660 lineage. One of the 2 samples (1/169; 0.6% [95% CI 0.0%–3.6%]) was also positive for the D/OK lineage by HI. Only the sample with double HI reactivity was also positive in the VN assay. For the samples collected in 2023, a total of 12 samples (12/257; 4.7% [95% CI 2.6%–8.1%]) were positive for D/660 lineage by HI, and none of the samples were positive for D/OK lineage (Table 2). The 2023 samples positive for HI were also tested in VN, and only 5 serum samples (41.7%) possessed neutralizing antibodies for D/660 lineage. The HI titers of the positive samples ranged from 10 to 160. The VN titers of the positive samples ranged from 10 to 80 (Table 2). Five of the 12 HI-positive samples tested positive for antibodies against ICV (Table 2).

**Table 1:** Serum testing for influenza D virus results, showing HI and VN assay titers of dog samples collected in 2016 in the Apulia region, Italy*

Sample no.	D/bovine/Oklahoma/660/2013			D/swine/Italy/199724–3/2015	
	HI	VN		HI	VN
4	10–10	<10		<10	<10
66	80–80	160–160		80–80	10–10

*Samples were tested against 2 strains of IDV, D/bovine/Oklahoma/660/2013 (D/660 lineage) and D/swine/Italy/199724–3/2015 (D/OK lineage). For calculation purposes, titers below the detectable threshold of 10 were expressed as <10. HI, hemagglutination inhibition assay; VN, virus neutralization assay.

**Table 2:** Serum testing for influenza D virus results showing HI and VN assay titers of dog samples collected in 2023 in the Apulia region, Italy*

Sample no.	D/bovine/Oklahoma/660/2013			D/swine/Italy/199724–3/2015			C/human/Victoria/2/2012
	HI	VN		HI	VN		HI
5	10–10	<10		<10	<10		40–40
18	10–10	20–20		<10	<10		<10
35	10–20	<10		<10	<10		<10
45	10–10	<10		<10	<10		<10
50	20–20	<10		<10	<10		20–10
55	40–40	10–10		<10	<10		80–80
98	10–10	<10		<10	<10		<10
118	10–10	20–20		<10	<10		40–40
137	160–160	80–80		<10	<10		<10
167	10–10	<10		<10	<10		80–80
183	20–20	<10		<10	<10		<10
216	20–20	20–20		<10	<10		<10

*Samples were tested against 2 strains of IDV, D/bovine/Oklahoma/660/2013 (D/660 lineage) and D/swine/Italy/199724–3/2015 (D/OK lineage), and against 1 strain of influenza C virus, C/human/Victoria/2/2012. For calculation purposes, titers below the detectable threshold of 10 were expressed as <10. HI, hemagglutination inhibition assay; VN, virus neutralization assay.

Our findings provide evidence of dog exposure to IDV at a low prevalence. Because most animals were household dogs, the source of exposure remains uncertain. Because our study was based on serologic investigations, any correlation between IDV infection and clinical signs in dogs could not be inferred. IDV infection in horses is subclinical, whereas in other animal species, IDV is associated with respiratory disease (*5*). Our findings are also partially in line with surveillance data from Europe and Italy suggesting the emergence of a D/660-like clade after the mid-2010s (*6*). In our study, none of the samples collected in 2023 reacted against D/OK, likely reflecting the epidemiology of IDV in susceptible animals in the Apulia region.

Although phylogenetic analyses have suggested that IDV is more closely related to ICV (≈53% homology of hemagglutinin-esterase fusion protein) than to influenza A and B viruses, no cross-reactivity was observed between IDV and human ICV by HI assay (*7*–*9*). To assess this potential issue caused by the genetic relatedness between IDV and ICV, the IDV-positive samples were also tested for ICV, and 5 samples did react against ICV. Four samples showed HI titers higher for ICV than for IDV, suggesting the possibility of cross-reactivity between IDV and ICV. Other serologic studies have investigated the antibody prevalence of IDV and ICV in animal populations (*8*–*10*), showing that serum reactivity in HI can vary among different IDV lineages and possibly between IDV and ICV. The combination of HI and VN assays may represent a good proxy of IDV circulation in animal hosts.

Our findings indicate that household dogs are exposed to IDV, potentially acting as a source of infection for humans. Although IDV does not seem to cause major forms of illness in humans and currently IDV is not regarded as an important public health threat, the possibility that some IDV strains may acquire the ability to evolve and adapt in the human host should not be disregarded. This potential risk could be higher in settings where there is more viral pressure, such as in groups with occupational exposure.

Appendix Additional information about detection of influenza D antibodies in dogs, Italy.
