# Supplementary material for: Detection of Influenza D Antibodies in Dogs, Apulia Region, Italy, 2016 and 2023
Source: Emerg Infect Dis. 2024 May;30(5):1045–7. doi: 10.3201/eid3005.231401 (PMC11060465; doi:10.3201/eid3005.231401)
Supplement: Appendix — Additional information about detection of influenza D antibodies in dogs, Italy. [file 23-1401-Techapp-s1.pdf]

Article DOI: https://doi.org/10.3201/eid3005.231401

EID cannot ensure accessibility for supplementary materials supplied by authors. Readers who have difficulty accessing supplementary content should contact the authors for assistance.

Detection of Influenza D Antibodies in Dogs, Apulia Region, Italy, 2016 and 2023Appendix

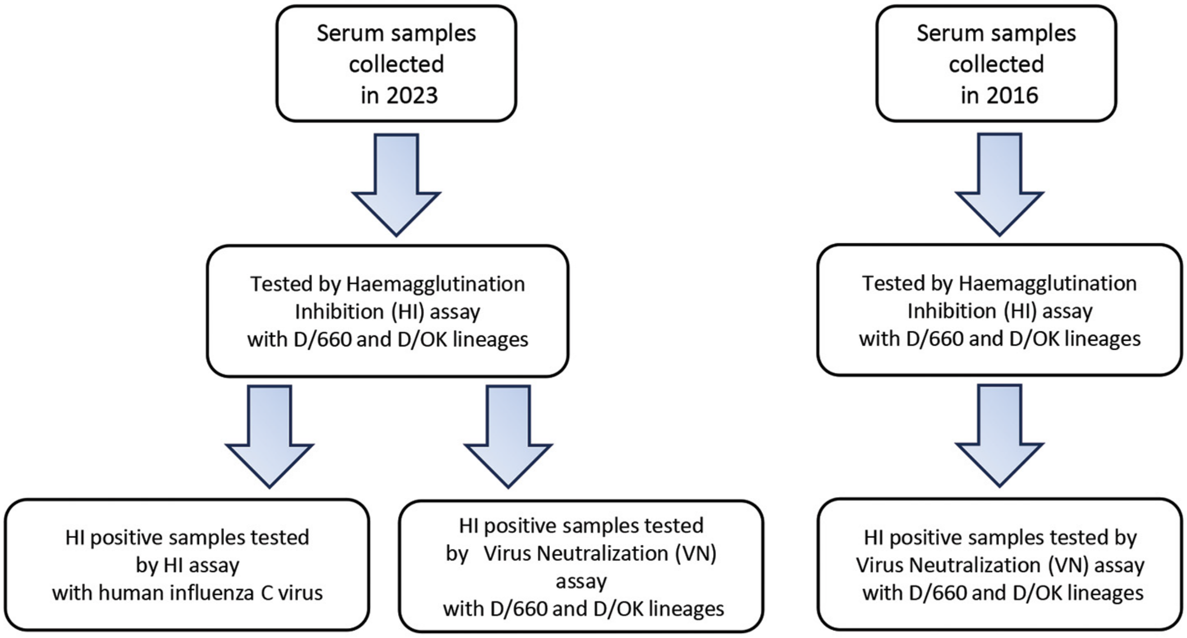

Appendix Figure. Workflow for serologic testing of dog samples collected in 2016 and 2023.
